# Gold nanoparticles: recent aspects for human toxicology

**DOI:** 10.1186/1745-6673-8-32

**Published:** 2013-12-11

**Authors:** Alexander Gerber, Matthias Bundschuh, Doris Klingelhofer, David A Groneberg

**Affiliations:** 1 Institute of Occupational-, Social- and Environmental Medicine, Goethe-University, Theodor-Stern-Kai 7, Haus 9b, 60590 Frankfurt am Main, Germany

**Keywords:** Gold, Auric nanoparticles, Toxicology, In-vivo

## Abstract

Nanoparticles (particles sized between 1 and 100 nanometers) are more and more used in all fields of science and medicine for their physicochemical properties. As gold has traditionally been considered as chemically inert and biocompatible, in particular, gold nanoparticles have been established as valuable tools in several areas of biomedical research. But in contrast to the multitude of studies that addressed the clinical use of gold nanoparticles, only little is known about potential toxicological effects such as induction of inflammatory immune responses, possible apoptotic cell death or developmental growth inhibition in embryos. Therefore the present study performed a systematic review of toxicological data, especially experimentally acquired data concerning in-vivo-toxicity, published in the PubMed. It can be stated that the data in this area of research is still largely limited. Especially, knowledge about size-, charge- and surface-chemistry dependent in-vivo-toxicity is needed to predict the hazard potential of auric nanoparticles (AuNPs) for humans.

## 

Nanoparticles, also called ultrafine particles, are defined as particles sized between 1 and 100 nanometers (10^-9^m) and form a bridge between bulk materials and atomic or molecular structures [1]. They occur in nature in the context of volcanic eruptions or any natural or anthropogenic combustion process. Man-made nanoparticles may appear for example as globular carbon molecule (fullerene or “buckyball”), as branched ribbons (dendrimers) or as nanotubes [2]. For gold has traditionally been considered inert and biocompatible, its physicochemical properties and high surface area, gold nanoparticles (AuNPs) are more and more used in biomedical research [3]. AuNPs of various sizes and morphologies have attracted considerable interest for medical applications for example as carrier for drugs such as paclitaxel [4], as tumor-detector [5], photothermal agent or radiotherapy dose enhancer (Figure 1) [6-8]. Nevertheless, experimental use of AuNPs presented possible medical hazards as the surface to volume ratio causes catalytic properties and can make particles very reactive [9]. Furthermore, Nanoparticles easily pass cell membranes and can interact with intracellular metabolism (Figure 2) [10]. As at nano-scale gold-particles may exhibit size-related properties that differ significantly from the known properties of non-nano-scaled gold-particles, one cannot predict reliably the nature of AuNPs and a biologic system and interactions between AuNPs and living cells [11]. Beside the size, further potentially toxic features of AuNPs depend on charge and surface-chemistry. To generate an overview of gold nanoparticle-induced toxicity, we performed a systematic review focused of toxicological data published in the PubMed.

**Figure 1:**
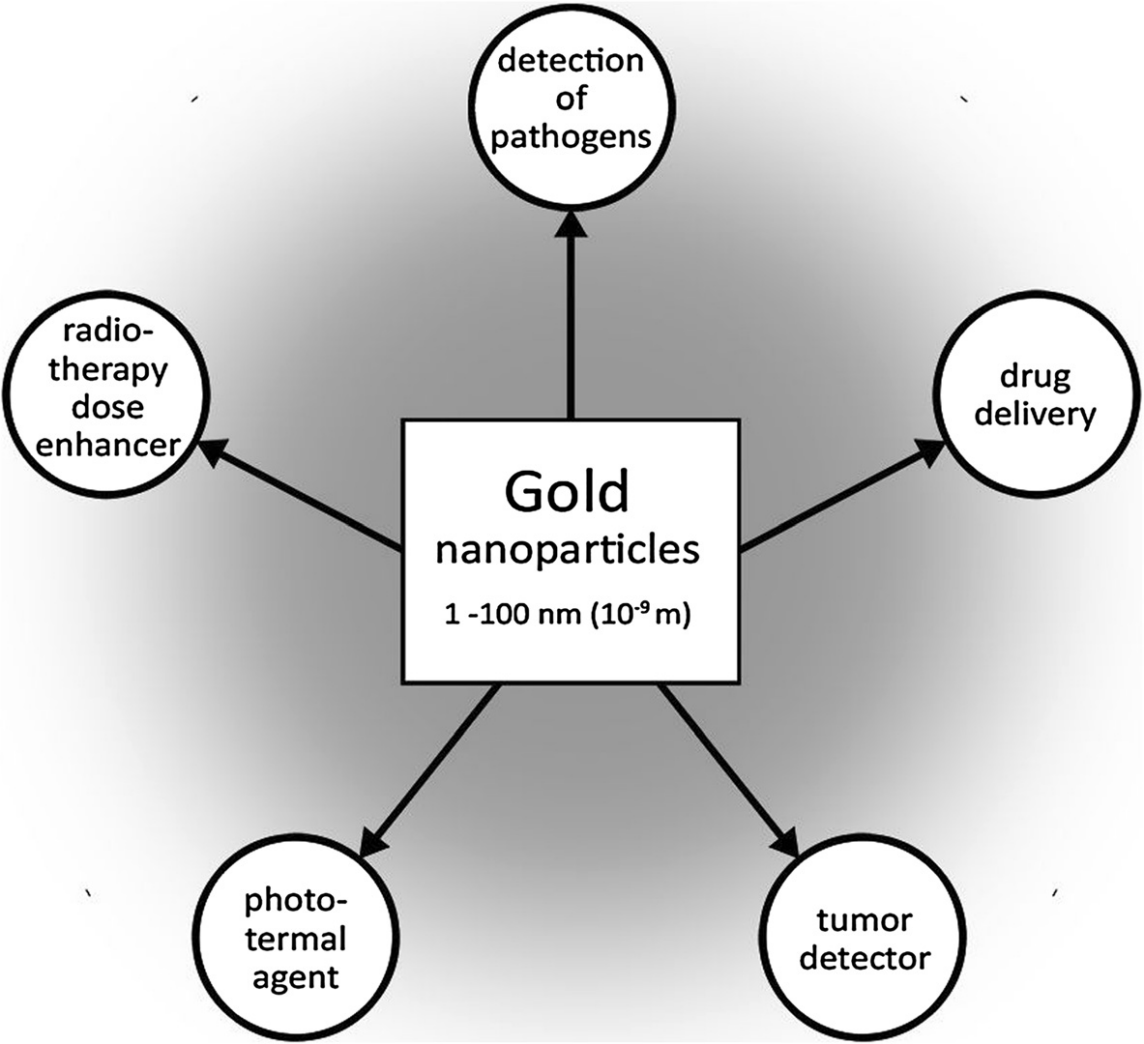
Common medical applications of gold nanoparticles.

**Figure 2:**
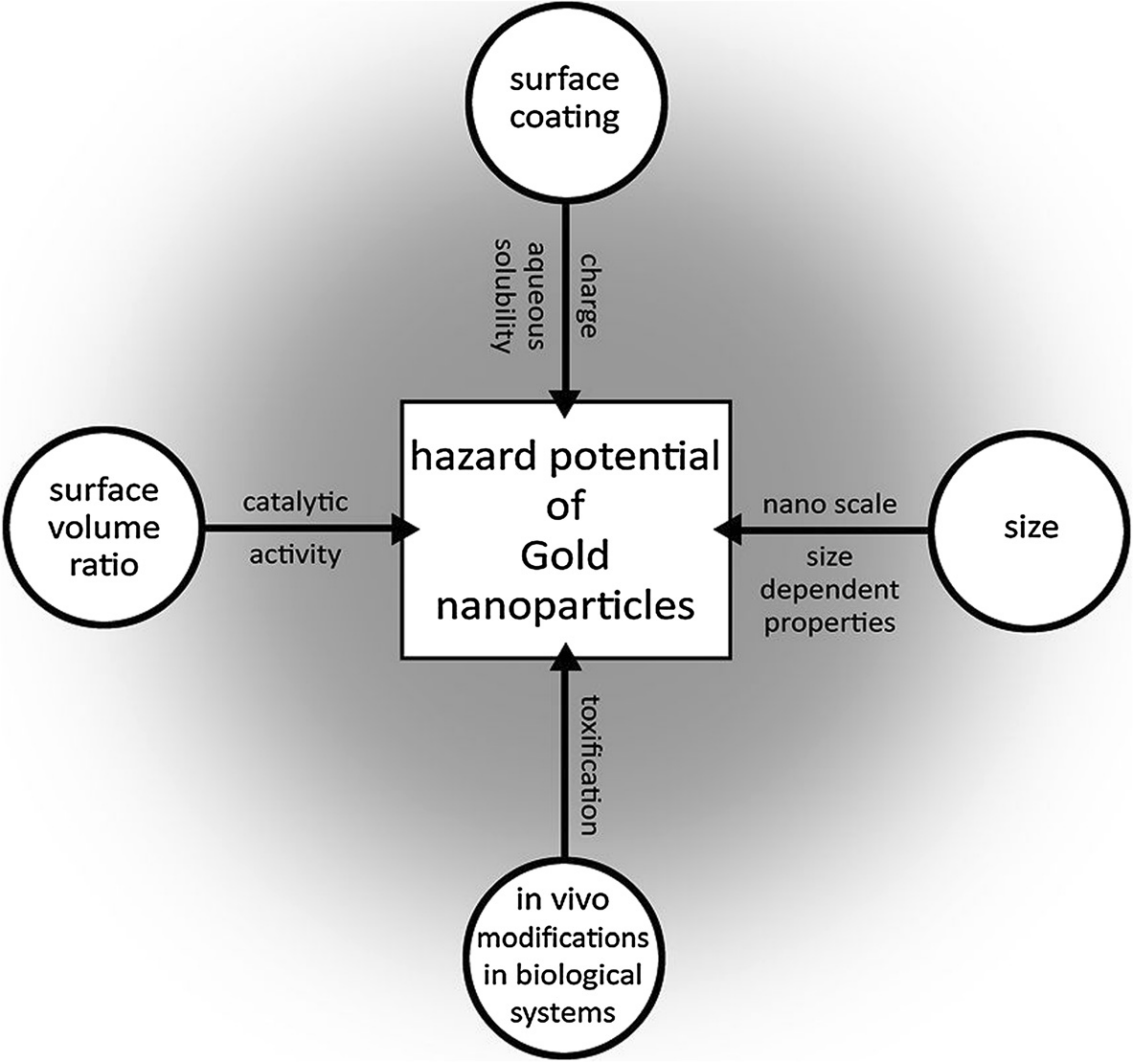
Aspects that contribute to the hazard potential of AuNPs.

In a recent study, Conde et al. [12] assessed gold nanoparticles (AuNPs) for aspects of genotoxicity and cell toxicity. The authors constructed an Antisense Gold-nanobeacon consisting of a stem-looped oligonucleotide double labeled with 3'-Cy3 and 5'-Thiol-C6 and tested for the effective blocking gene expression in colorectal cancer cells. They also studied this system for the proteomic effects of gold-nanobeacon exposure to cancer cells. Exposure was evaluated by two-dimensional protein electrophoresis followed by mass spectrometry to perform a proteomic profile and MTT assay, Glutathione-S-transferase assay, micronucleus test and comet assay to assess the genotoxicity [12]. Overall, the authors concluded that the proposed nanoparticle strategy does not exhibit significant toxicity [12]. Upon studies in vitro have demonstrated, that surface chemistry plays a crucial role in determining toxicity of AuNPs [13], Kim et al. lately examined the role of surface charge and size on AuNPs in in-vivo toxicity using an embryonic zebrafish model and found out that the surface functionalization dictated toxicity outcomes with embryos [14]. In this study, exposure of zebrafish embryos to 1.3 nm AuNPs functionalized with a monolayer of the cationic ligand, N,N,N-trimethylammoniumethanethiol (TMAT-AuNPs), emerged as highly developmentally toxic, causing embryo lethality and numerous morphological effects as abnormally small and underpigmented eyes [14]. Exploring the mechanism underlying this effect, the scientists determined that TMAT-AuNPs caused a significant increase of apoptotic cell death in the eye and aberrant expression of transcript factors that regulate eye- and pigmentation development (pax6a, pax6b, otx2, and rx1) and pigmentation (sox10). Embryos exposed to sublethal concentrations of TMAT-AuNPs showed hypoactivity and axonal growth inhibition. The authors come to the conclusion that TMAT-AuNPs may pose a developmental hazard to mammals [14]. Also using an embryonic zebrafish model, Truong et al., from the Oregon State University, investigated how surface functionalisation and charge of AuNPs influence molecular responses in vivo, utilizing dechorionated embryonic zebrafishs, exposed to AuNPs from 6 to 24 or 6 to 48 h post fertilization [15]. The authors used precisely engineered AuNPs with 1.5 nm cores and functionalized with three ligands: 2-mercaptoethanesulfonic acid (MES), N,N,N-trimethylammoniumethanethiol (TMAT), or 2-(2-(2-mercaptoethoxy)ethoxy)ethanol. The scientists confirmed AuNP uptake in exposed embryos using inductively coupled plasma-mass spectrometry. Developmental assessments revealed differential biological responses for each NP type when embryos were exposed to the functionalized AuNPs at the same concentration [15]. TMAT-AuNPs were lethal to embryos, MES-AuNPs induced sublethal malformations and MEEE-AuNPs did not induce any in vivo biological response. Effects of MES- and TMAT-AuNPs in exposed embryonic zebrafishs included inflammation, immune response and misregulation of transport mechanisms. The authors conclude that surface functionalization of AuNPs influences the biological response at the phenotypical and molecular levels.

The long-term effect of exposure to AuNPs during embryonic development was investigated also by Lisa Truong et al. in a zebrafish model one year ago [16]. In this study, the scientists wanted to identify whether acute exposure to negatively charged 2-mercaptoethanesulfonic acid- (MES), or neutral 2-(2-(2-mercaptoethoxy)ethoxy)ethanol- (MEEE) or positively charged trimethylammoniumethanethiol- (TMAT) AuNPs would lead to deleterious effects that persist into adulthood and specifically, whether the charged surface functional groups would impact development of the central nervous system leading to abnormal behavior or survivorship in adulthood. Therefore zebrafish embryos were acutely exposed to the three gold NPs with differing surface charge. The scientists found out that both MES- and TMAT-AuNP exposed embryos exhibited hypo-locomotor activity, while those exposed to MEEE-AuNPs did not. Evaluation of behavioral abnormalities and the number of survivors at 122 days post fertilization showed that both treatments induced abnormal startle behavior following a tap stimulus [16]. But the group exposed to the negatively charged MeS-AuNPs also exhibited abnormal adult behavior in the light and had a lower survivorship into adulthood. The authors sum up that exposure to NPs differing only in the functional group, affects larval behavior, with behavioral effects persisting into adulthood [16]. Using Drosophila melanogaster as model organism, another study is also concerned with the long-term genotoxic and mutagenic effects of AuNPs in vivo. Vecchio et al. from the Italian Institute of Technology, have shown in a recent work that exposure to AuNPs may lead to signigicant phenotypic modifications in Drosophila melanogaster which even may be transmitted to the progeny [17]. Via treatment of Drosophila melanogaster for an entire life-cycle (eggs-to-eggs) with citrate-capped 15 nm AuNPs which were formulated in the diet at a dose of NP to the flies 3 μg/g per day, the scientists obtained the first nanomaterial-mutated organism, named NM-mut. Increased levels of reactive oxygen species are discussed to be the underlying mechanism for the observed variations in the genome-wide expression profile induced by AuNPs [18]. Compared with the descendants of non-treated organisms, the mutated flies reveal impaired fecundity and fertility, as well as morphological defects of the wings an of the eyes. The Authors conclude that 15 nm AuNPs are able to induce genetic mutations in the germinal line of Drosophila that may be transmitted to the progeny [15]. In another recent publication concerning the surface-dependend toxicity of AuNPs, Fraga et al. investigated the cytotoxic effects of AuNPs in human liver HepG2 cells. The Portugal scientists utilized ~20 nm spherical AuNPs (0-200 μM Au) with two surface coatings (citrate (Cit) compared with 11-mercaptoundecanoic acid (11-MUA)) [19] and evaluated cytotoxicity, via 3-(4,5-dimethylthiazol-2-yl)-2,5-diphenyltetrazolium bromide (MTT) reduction and lactate dehydrogenase (LDH) release assays after 24 to 72 h of incubation and assessing DNA damage by the comet assay, 24 h after incubation with the capped AuNPs. Quantification by graphite furnace atomic absorption spectrometry and transmission electron microscopy revealed that both AuNPs were internalized in a concentration-dependent manner and no differences were found in the extent of the internalization between the two types of NPs. Furthermore, both differently coated AuNPs did not induce significant cytotoxicity [19]. But in spite of absent cytotoxicity, the authors observed genotoxic effects of Cit-AuNPs, namely inversely proportional to the tested concentrations, which did not occur in MUA-AuNPs-exposed cells [19]. In summary, the authors point out the importance of the surface properties to increase the biocompatibility and safety of AuNPs. AuNP-mediated hepatotoxicity in mice with healthy or damaged livers was examined by Hwang et al. in a recent study in south Korea, using a mouse model of nonalcoholic steatohepatitis (NASH) [20]. The searchers induced a model of liver injury by feeding mice with a methionine- and choline-deficient (MCD) diet for 4 weeks. After lateral tail vein injection with 5mg/kg 15-nm PEGylated AuNPs in MCD-diet-fed mice and normal- fed mice, sizes and biodistribution of the AuNPs were analyzed by transmission electron microscopy, levels of alanine aminotransferase (ALT) and aspartate aminotransferase (AST) were estimated with an automatic chemical analyzer, activities of antioxidant enzymes were determined by biochemical assay and liver sections were subjected to pathological examination. The authors observed that AuNPs significantly elevated the serum ALT and AST levels in MCD diet-fed mice compared to MCD diet-fed mice injected only with mPEG (methylpolyethylene glycol). Furthermore, severe hepatic cell damage, acute inflammation, and increased apoptosis and reactive oxygen species (ROS) production were observed in the livers of AuNP-injected mice on the MCD diet [20]; whereas these liver injuries were attenuated in mice fed a normal chow diet. The authors suggest that AuNPs display toxicity in a stressed liver environment by stimulating the inflammatory response and accelerating stress-induced apoptosis [20]. They recommend considering health conditions, including liver damage, in medical applications of AuNPs.

The importance of understanding electrostatic interactions between charged nanoparticles and monolayers as model membranes to predict interactions between nanoparticles and living cells in the future, was quite lately considered also in Portugal by Torrano et al. [21]. The authors showed how oppositely charged gold nanoparticles (Au-NPs) interact with monolayers of the zwitterionic dipalmitoylphosphatidyl choline (DPPC) and negatively charged dipalmitoylphosphatidyl glycerol (DPPG). For this purpose they spread monolayers on subphases containing two concentrations of either negatively charged Au-NPs coated with citrate anions or positively charged Au-NPs functionalized with the cationic polyelectrolyte poly(allylamine hydrochloride) (PAH). The charged nanoparticles had remarkable effects on DPPG monolayers which were obviously larger for the positively charged AuNPs. But negatively charged ones also affected the monolayer properties owing to the influence of counter ions. The in-plane elasticity for DPPG monolayers within the surface pressure range corresponding to real cell membranes increased with adsorption of positively charged NPs, but decreased with the negative ones. For the zwitterionic DPPC, on the other hand, significant effects only occurred for negatively charged NPs, including a decrease in elasticity [21]. The authors discussed the dependence of the different parameters, capping of nanoparticles and type of monolayer, explaining why toxicity of a given nanoparticle cannot be easily predicted and came to the conclusion that namely the charge of the capping agents is crucial for the interaction of charged NPs with the cell membrane [21]. They emphasize that more systematic studies probing not only electrostatic but also hydrophobic and other types of interaction are needed to better predict toxicity of nanoparticles in future. The functional impact of Au-NPs on B-lymphocytes, was investigated by Sharma M et al., using a murine B-lymphocyte cell line (CH12.LX), [22]. The scientists treated B-lymphocytes with citrate-stabilized 10 nm Au-NPs and observed activation of an NF-κB-regulated luciferase reporter, which correlated with altered B lymphocyte function (i.e. increased antibody expression). They suppose that Au-NPs could interact with intracellular components of the NF-κB signaling pathway after passed through the cellular membrane, which was shown by TEM imaging. To support this theory, Sharma et al. showed, using immune-electrophoresis, that IKKα and IKKβ can bind specifically to Au-NPs, based on their inherent property to bind to –thiol groups, when CH12.LX lysate is exposed to 10 nmAu-NPs [22]. The authors discuss that altered NF-κB signaling and cellular function in B-lymphocytes suggests a potential for off-target effects with in vivo applications of gold nanomaterials. Tsyusko et al., an US-American group of scientists from Lexington, Kentucky, examined the in vivo particle-specific genomic toxicity of Au-NPs to Caenorhabditis elegans [23]. Therefor the scientists exposed nematodes to 4-nm citrate-coated Au-NPs at a concentration resulting in 10% mortality (5.9 mg/L). Analysis of the toxicogenomic response via whole genome microarray, they identified significant differential expression of 797 genes. The authors independently confirmed the levels of expression for five genes (apl-1, dyn-1, act-5, abu-11, and hsp-4) with qRT-PCR and identified seven common biological pathways associated with 38 of these genes [23]. They observed up-regulation of 26 pqn/abu genes from noncanonical unfolded protein response (UPR) pathways and molecular chaperones (hsp-16.1, hsp-70, hsp-3, and hsp-4) which are likely indicative of endoplasmic reticulum stress. Furthermore Tsyusko et al. discuss involvement of the genes from this pathway in a protective mechanism against Au-NPs, as a mutant from noncanonical UPR (pgn-5) shows increased sensitivity to Au-NPs. The finding that endocytosis mutants (chc-1 and rme-2) significantly respond to Au-NPs was interpreted as evidence for endocytosis pathways being induced by Au-NPs. The authors concentrate their observations mentioning Au-NPs causing adverse effects to C. elegans by activating both general and specific biological pathways and suppose, several of these pathways being involved in Au-NP toxicity and/or detoxification.

In another recent study to in-vivo-toxicity of AuNPs, Perreault et al. investigated the toxicity of generation 0 PAMAM-coated gold nanoparticles (AuG0 NPs) in four different biological models to determine how different cellular systems are affected by PAMAm-coated NPs [24]. The most common mechanisms by which PAMAM dendrimers are thought to induce toxicity are membrane disruption [25] and formation of reactive oxygen species [18]. Therefore Perreault et al. evaluated toxicity in two mammalian cell lines (Neuro 2A and Vero), in the green alga Chlamydomonas reinhardtii and the bacteria Vibrio fischeri. The scientists observed that AuG0 NP treatments reduced cell metabolic activity in algal and bacterial cells, measured by esterase enzymatic activity (C. reinhardtii) and luminescence emission (V. fischeri) [24]. Contrariwise, almost no toxicity was observed in mammalian cells after treatment to AuG0 NPs. The authors interpret the observed low toxicity of AuG0 Nps in mammalian cells compared with the more sensitive algal and bacterial cells on the one hand due to their specific cellular structure and specific properties (i.e. higher amphiphilicity and fluidity) and on the other hand with possible modifications of the surface properties of NPs in the cell culture medium, used for the mammalian cell lines [24]. The authors recommend taking these observations into account when designing PAMAM NPs for applications that may lead to their introduction in the environment. To investigate lung toxicity of airborne nanomaterials and its dependency on particle size, Schulz et al. have investigated the genotoxic effects of 18 μg gold nanomaterials of the same composition, but different sizes (2,20 and 200 nm) administered by single intratracheal instillation into the lung of male adult Wistar rats [26]. Chosen endpoints of this study were alkaline Comet assay in lung tissue and micronucleation in polychromatic erythrocytes of the bone marrow 72 h after single instillation. The authors could not detect relevant DNA damage in the mentioned tests. Furthermore the measurement of clinical pathology parameters in bronchoalveolar lavage fluid (BALF) and blood indicated neither relevant local reactions in the animals' lungs nor adverse systemic effects. The scientists come to the conclusion that under the conditions of this study the different sized AuNPs tested were non-genotoxic and showed no systemic and local adverse effects at the given dose [26]. In another work regarding size- dependent in vivo toxicity of AuNPs, Zhang et al. investigated the effects of 5, 10, 30 and 60 nm PEG-coated gold nanoparticles in mice [27]. Therefore the mice received an intraperitoneal injection of approximately 200 μL of AuNPs solution at a dose of 4000 μg/kg. 28 days after administration, the scientists evaluated animal survival, bodyweight, biodistribution, bloodchemistry, biochemistry and characteristics on transmission electron microscopy. Neither an obvious decrease in body weight could not be detected, nor appreciable toxicity. But the authors observed that Accumulation of AuNPs in different organs was size dependent. 5 nm and 10 nm particles preferentially accumulated in the liver and 30 nm particles in the spleen. Accumulation of 5, 10, 30 and 60 nm particles in the blood and bone marrow was proved by transmission electron microscopy. Spleen index and thymus index were increased, probably due to an affection of the immune system by small nanoparticles. Whilst the 10 nm gold particles induced an increase in white blood cells, 5 nm and 30 nm particles induced a decrease in white blood cells and red blood cells. A significant increase in alanine transaminase and aspartate transaminase levels was caused by the 10 nm and 60 nm PEG-coated AuNPs, indicating a damage to the liver. The authors conclude that in vivo toxicity of PEG-coated gold particles in mice is complex but not strictly size-dependent. The toxicity of 10 nm and 60 nm particles was higher than that of 5 nm and 30 nm particles [27].

## Conclusion

This review of the recent published literature shows that the potential toxic impact of AuNPs may be multisided and is hard to predict. Beside particles size, also surface chemistry and charged surface functional groups play a crucial role in determining genotoxic-, mutagenic- or cell toxic effects. Hence experimentally acquired knowledge about size-dependent toxic effects, surface properties of AuNPs and their eventually expected modifications in biological systems are essential to predict the impacts of AuNPs in vivo and to reduce the hazard potential for humans in the long run.

## Competing interests

The authors declare that they have no competing interests.

## Authors’ contributions

DK, DAG and MB made substantial contributions to the conception and design of the review, acquisition of the review data and have been involved in drafting and revising the manuscript. All authors have read and approved the final manuscript.
